# Analysis of acid-tolerance mechanism based on membrane microdomains in *Saccharomyces cerevisiae*

**DOI:** 10.1186/s12934-023-02195-y

**Published:** 2023-09-13

**Authors:** Xueqin Lv, Ke Jin, Yu Yi, Lingang Song, Xiang Xiu, Yanfeng Liu, Jianghua Li, Guocheng Du, Jian Chen, Long Liu

**Affiliations:** 1 Key Laboratory of Carbohydrate Chemistry and Biotechnology, Ministry of Education, Jiangnan University, Wuxi, 214122 China; 2 Science Center for Future Foods, Jiangnan University, Wuxi, 214122 China; 3 Yixing Institute of Food Biotechnology Co., Ltd, Yixing, 214200 China; 4 Food Laboratory of Zhongyuan, Jiangnan University, Wuxi, 214122 China

**Keywords:** *Saccharomyces cerevisiae*, Acid-tolerance mechanism, Membrane microdomain, H^+^-ATPase

## Abstract

**Background:**

*Saccharomyces cerevisiae* has been used in the biosynthesis of acid products such as organic acids owing to its acid tolerance. Improving the acid tolerance of *S. cerevisiae* is beneficial for expanding its application range. Our previous study isolated the TAMC strain that was tolerant to a pH 2.3 through adaptive laboratory evolution; however, its mechanism underlying tolerance to low pH environment remains unclear.

**Results:**

In this study, through visual observation and order analysis of plasma membrane and membrane microdomains, we revealed that the membrane microdomains of TAMC strain play an indispensable role in acid tolerance. Transcriptomic analysis showed an increase in the expression of genes related to key components of membrane microdomains in TAMC strain. Furthermore, an obvious reduction was observed in the acid tolerance of the strain with sterol C-24 methyltransferase encoding gene *ERG6* knockout for inhibiting membrane microdomain formation. Finally, colocalization analysis of H^+^-ATPase PMA1 and plasma membrane protein PMP1 showed that disruption of membrane microdomains could inhibit the formation of the H^+^-ATPase complex.

**Conclusions:**

Membrane microdomains could provide a platform for forming H^+^-ATPase complexes to facilitate intracellular H^+^ homeostasis, and thereby improve cell acid resistance. This study proposed a novel acid tolerance mechanism, providing a new direction for the rational engineering of acid-tolerant strains.

**Supplementary Information:**

The online version contains supplementary material available at 10.1186/s12934-023-02195-y.

## Introduction

*Saccharomyces cerevisiae* is one of the main microbial chassis for bioproduction owing to its short growth cycle, high fermentation ability, and good performance at large-scale [1–4]. It is important to increase the tolerance of the microbial chassis based on the desired product and environment [5, 6]. Recently, researchers have focused on improving the tolerance of *S. cerevisiae* to various stresses, such as exposure to high temperatures, acids, alkalis, and alcohols, to improve its robustness in different environments [7–10]. Acid stress is the most common stress encountered by yeast cells during fermentation, especially during the fermentation of acid products such as organic acids [11, 12]. It is often necessary to add a large number of neutralizers to alleviate the inhibition of acid stress on cell growth and product synthesis [13], which not only makes the downstream purification process complex, but also easily causes environmental pollution.

To circumvent the abovementioned issues, researchers have attempted to isolate acid-tolerant strains through adaptive laboratory evolution (ALE), and achieved remarkable success. For example, Pereira et al. obtained *S. cerevisiae* mutants tolerant to coumaric acid or ferulic acid (pH 3.5) using ALE and revealed that improvement in the tolerance of those mutant strains was mainly related to the activity regulation of the aromatic acid transporter *ESBP6* [12]. Jang et al. isolated an acid-tolerant (pH 4.2) *S. cerevisiae* mutant, which showed a 17% increase in the L-lactic acid titer to 119 g/L [14]. However, the pH of fermentation media in the industrial bioproduction is often < 3.0, indicating that the reported strains resistant to weak acid (pH 3.5–5.8) cannot meet the preferred bioproduction requirements. To develop an acid-tolerant *S. cerevisiae* that can tolerate a pH of < 2.5, using L-malic acid and citric acid as screening pressure, we finally isolated a strain named TAMC with tolerance to a pH of 2.3 after ~ 1,700 generations of evolutionary screening [15]. However, the specific acid-resistant mechanism requires further investigation.

To date, several mechanisms have been reported to be involved in acid-tolerance regulation, including the activity of proton pumps or ATP-binding cassette (ABC) transporters, intracellular pH homeostasis, cell wall remodeling associated with glycosylphosphatidylinositol (GPI)-anchored protein, and changes in plasma membrane ergosterol levels [12, 16–18]. For example, H^+^-ATPase PMA1, a proton pump accounting for 15% of the total membrane proteins, can pump protons out of the cell, which is essential for regulating intracellular pH [19]. Interestingly, PMA1, GPI-anchored proteins, and ergosterol are closely related to membrane microdomains rich in sterols and sphingomyelin and can be used as biological platforms for protein anchoring. PMA1 and GPI-anchored proteins are resident proteins, and ergosterol is the key sterol [20–22]. Five typical membrane microdomains are present in *S. cerevisiae*: MCP (H^+^-ATPase PMA1), MCC (membrane compartment of Can1), MCT (TORC2 kinase), MCL (sterol transporters Ltc3/4), and MCW (cell wall stress mechanosensor Wsc1) [21, 23]. MCP is important for maintaining intracellular H^+^ homeostasis and is involved in endocytosis, secretion, and cell wall synthesis. MCC is related to the cell’s response to sudden changes in the alkaline pH environment [24]. Therefore, it was speculated that membrane microdomains are important for acid tolerance in *S. cerevisiae*; however, this finding has not yet been confirmed.

In this study, taking *S. cerevisiae* TAMC as the research object, through visual observation and transcriptomic analysis, we revealed that membrane microdomains play an indispensable role in the acid-tolerant mechanism. When ergosterol synthesis was inhibited to suppress the formation of membrane microdomains, the acid resistance of the strain was significantly inhibited. Further colocalization analysis revealed that membrane microdomains could provide a platform for forming H^+^-ATPase complexes to facilitate intracellular H^+^ homeostasis. This study proposed a novel acid-tolerance mechanism and provided a new direction for the rational engineering of acid-tolerant strains.

## Results and discussion

### Analysis of cell growth and subcellular characterization of acid-tolerant strains

**Fig. 1:**
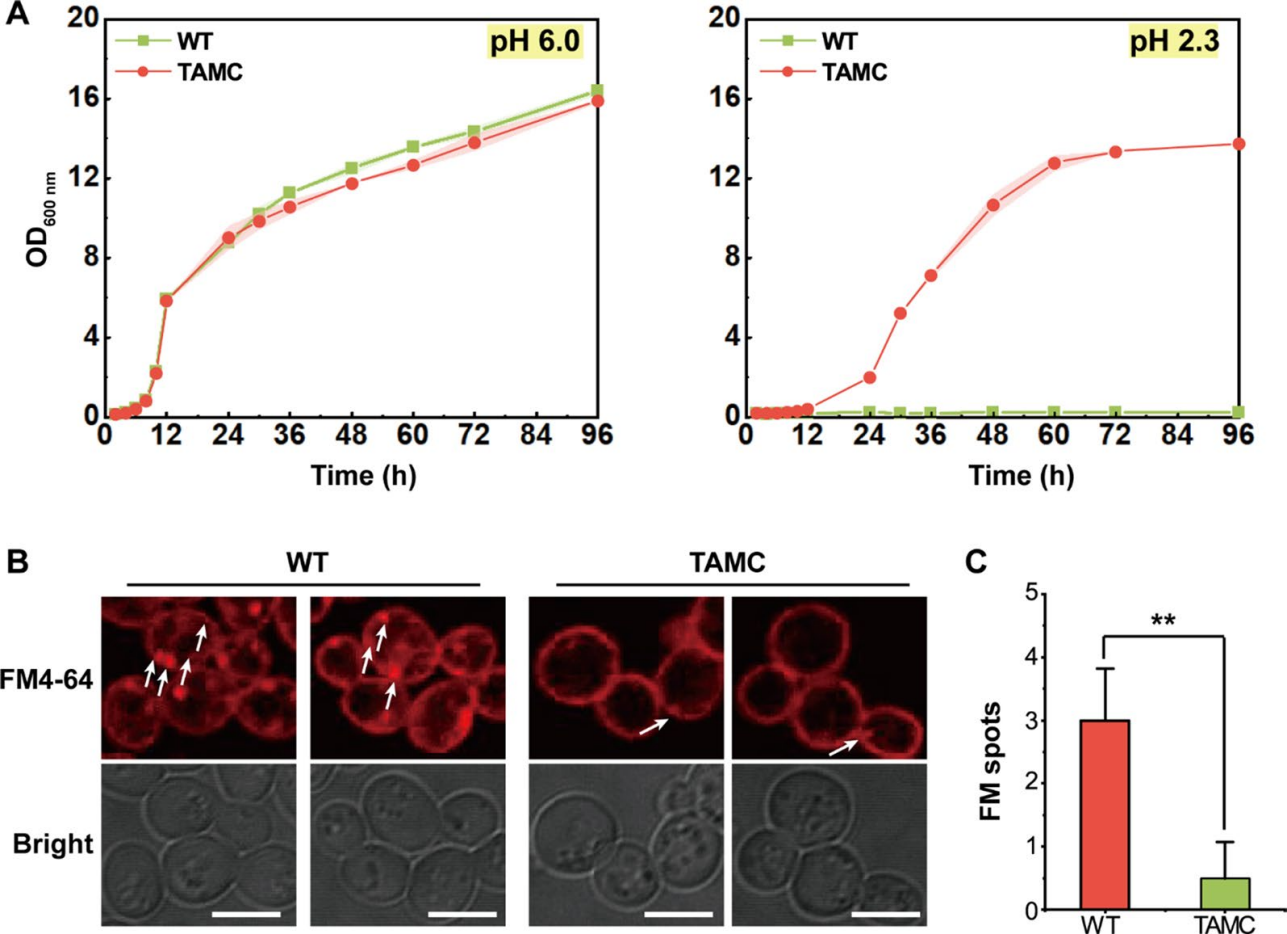
Phenotypic analysis of WT and TAMC strains in YPD medium at pH 6.0 and 2.3. **A** Changes in biomass (OD_600 nm_) of WT and TAMC strains in YPD medium at pH 6.0 and 2.3 during fermentation. **B** FM4-64 images of WT and TAMC strains in YPD medium at pH 6.0. The white arrows indicate the FM fluorescent plots. Scale bar, 5 µm. **C** FM fluorescent plots of WT and TAMC strains

To thoroughly examine the characteristics of the acid-resistant strain TAMC, cell growth, cell integrity, and intracellular vesicles were analyzed. Using L-malic acid, the pH of YPD medium was adjusted to 6.0 and 2.3. During the whole culture period, the TAMC strain showed good growth status as the WT strain at pH 6.0. In contrast, at pH 2.3, the growth of the WT strain was significantly inhibited, but the TAMC strain showed similar growth status as that at pH 6.0. The maximum OD_600 nm_ reached 13.7 at pH 2.3 (Fig. 1A). Further, SEM analysis revealed that the morphology of the TAMC strain was extremely similar to that of the WT strain cultured in YDP media (pH 6.0). In contrast, in pH 2.3 media with, compared with the normal morphology of TAMC cells, some WT cells were oval, with more severe lysis (Additional file 1: Fig. S1).

The plasma membrane is an important barrier for cells to cope with adverse environments. Accordingly, we next observed plasma membrane integrity. The membrane-selective fluorescent dye FM4-64 was used to stain the membrane structure and follow membrane internalization of TAMC and WT strains. As shown in Fig. 1B, the plasma membrane of the TAMC strain was intact and unperturbed, similar to that of the WT strain. However, a significant difference in the number of FM4-64 fluorescence spots internalized into the cells was observed between the TAMC and WT strains (Fig. 1B). The number of FM4-64 fluorescence spots in the observation fields was 0–1 per cell in TAMC strain and 3–4 per cell in WT strain in pH 6.0 medium (Fig. 1C). Based on results, it can be inferred that the acid resistance of this strain is probably closely related to the intracellular transport process, which is precisely dependent on membrane microdomains such as MCP and MCT [23]. In addition, membrane microdomains are closely related to the acid or alkaline environments [21, 24]. We next focused on exploring the characteristics of membrane microdomain distribution in acid-tolerant and control strains.

### Visual analysis of the correlation between membrane microdomains and acid tolerance

**Fig. 2:**
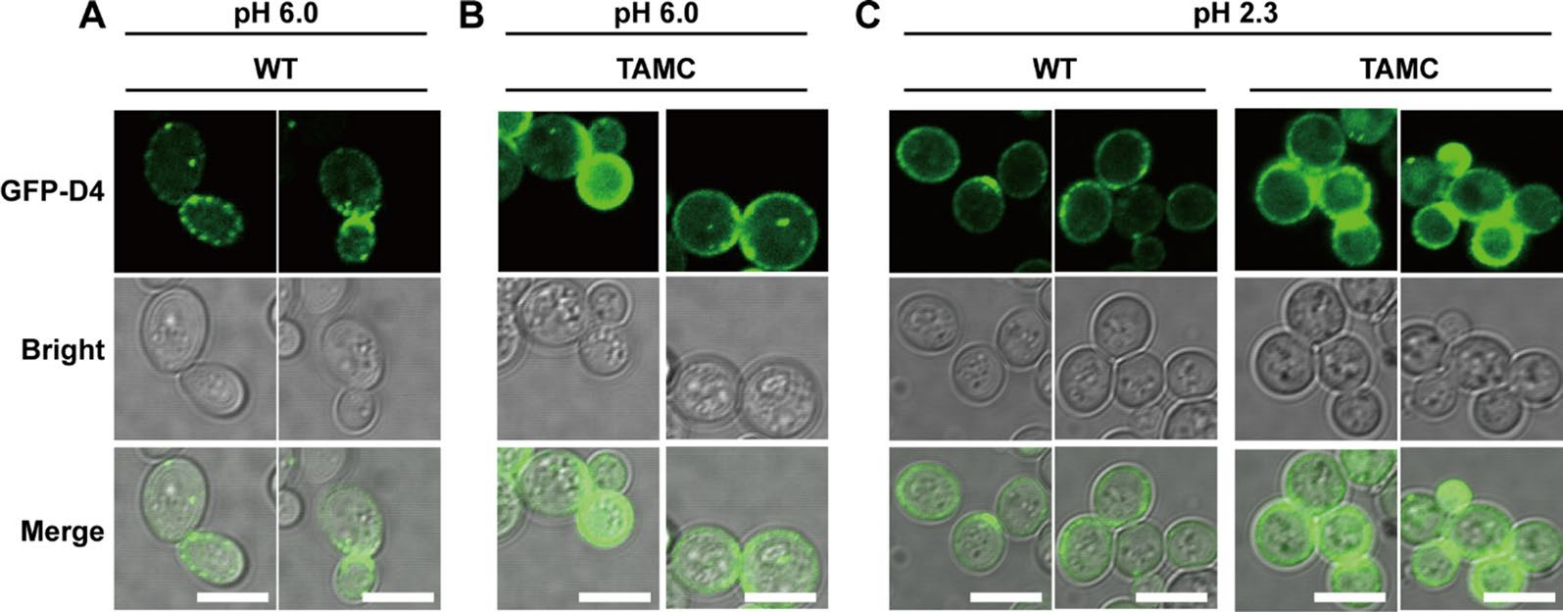
Localization of GFP–D4 in WT and TAMC strains in different pH media. **A** Localization of GFP–D4 in WT strain in YPD medium at pH 6.0. **B** Localization of GFP–D4 in TAMC strain in YPD medium at pH 6.0. **C** Localization of GFP–D4 in WT and TAMC strains in YPD medium at pH 2.3. The first line represents the GFP–D4 image, the second line represents the bright-field images, and the third line represents the superposition of GFP–D4 and bright-field images. Scale bar, 5 µm

Domain 4 (D4), the C-terminal domain of perfringolysin O, can specifically bind to cholesterol, thereby serving as a probe for lipid rafts in mammalian cells [25]. To determine whether D4 can specifically label membrane microdomains, a recombinant strain expressing the green fluorescent protein (GFP)-D4 fusion protein was constructed using *S. cerevisiae* CEN.PK2-1C as the host. Confocal imaging showed that GFP-D4 exhibited a discontinuous punctate distribution on the cell membrane (Fig. 2A), consistent with typical localization characteristics of membrane microdomains [21], suggesting that GFP-D4 can specifically label membrane microdomains in *S. cerevisiae*. Therefore, a visual analysis of membrane microdomains in WT and TAMC strains was performed using GFP-D4. Unlike the punctate distribution of GFP-D4 expression in the WT strain, GFP-D4 expression in the TAMC strain was higher on the plasma membrane, making it difficult to observe a clear punctate distribution (Fig. 2B). After transferring the WT strain from pH 6.0 to 2.3 medium for 10 h, GFP-D4 expression on the plasma membrane of the WT strain showed an evident increase, exhibiting similar distribution characteristics as the TAMC strain (Fig. 2C). Similarly, when TAMC strains were cultured in pH 2.3 medium, the distribution of GFP-D4 on the plasma membrane would further increase (Fig. 2C). Considering that *S. cerevisiae* can reduce the potential toxicity caused by weak acids through its stress response [26], it was speculated that GFP-D4 labeled membrane microdomains are involved in the stress process. Thus, when the WT strain was cultured in pH 2.3 medium for 10 h, the varying trend of GFP-D4 expression was similar to that of the acid-tolerant TAMC strain.

**Fig. 3:**
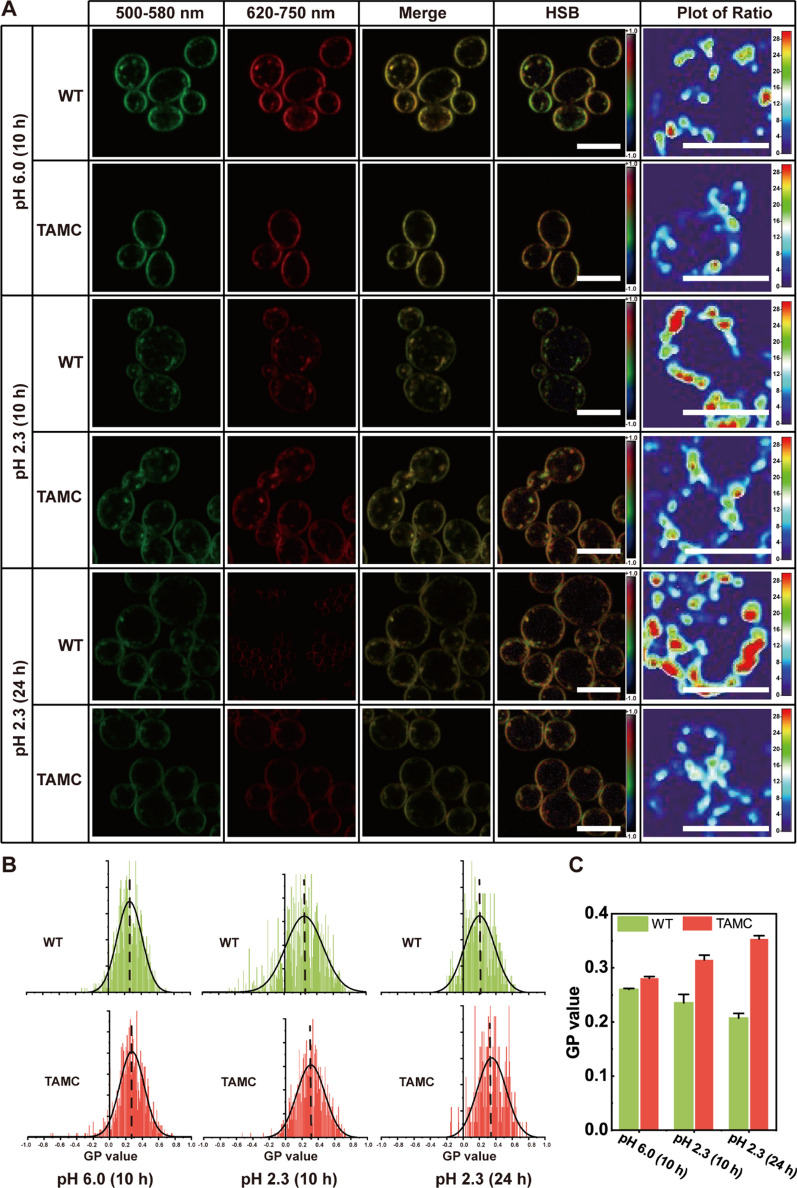
Analysis of membrane order degree in WT and TAMC strains. **A** Confocal dual-channel imaging of WT and TAMC strains labeled using di-4-ANEPPDHQ. The first and second left images indicate green (500–580 nm) and red (620–750 nm) channel images, respectively; the third middle image represents the superposition of red and green channels; the second right image is the HSB image; and the first right image is the surface plot of the ratio. The first and second lines are the confocal dual-channel imaging of TAMC and WT strains in YPD medium at pH 6.0, respectively. The third and fourth lines are the confocal dual-channel imaging of TAMC and WT strains in YPD medium at pH 2.3. Scale bar, 5 μm. **B** Histogram of GP value distribution of the membranes of WT and TAMC strains in YPD medium at pH 6.0 and 2.3. **C** GP value of the membranes of WT and TAMC strains in YPD medium at pH 6.0 and 2.3. Scale bar, 5 µm

Next, we used di-4-ANEPPDHQ, a phase-sensitive membrane probe that can quantify the membrane order [27], to quantify changes in membrane microdomains during the acid resistance process. In general, the emission wave of the lipid disordered phase is 620–750 nm, whereas that of the lipid ordered phase is 500–580 nm; thus, in the lipid ordered phase, the emission spectra of di-4-ANEPPDHQ exhibits a blue shift [28]. Based on this, a ratio-based method was used to visualize the degree of membrane ordering. As shown in Fig. 3A, compared with the WT strain, the emission spectra of di-4-ANEPPDHQ exhibited a blue shift in the TAMC strain. However, when the pH of the medium decreased to 2.3 (the strains were first cultured in normal YDP medium for 24 h, and then transferred to acidic medium at a 10% inoculation rate), with the extension of incubation time, the emission spectrum of di-4-ANEPPDHQ of the WT strain gradually exhibited a red shift, while that of the TAMC strain gradually showed a blue shift. The GP value analysis showed that the order degree of the TAMC strain in normal YPD medium was 0.28. After being cultured in pH 2.3 medium for 10 h, the order degree increased to 0.31. As the culture time increased, the order degree also increased, reaching the highest order degree of 0.35 at 24 h (Fig. 3B,C). In contrast, the order degree of the WT strain in normal YPD medium was 0.26. After being cultured in pH 2.3 medium for 10 h, the order degree decreased to 0.24. As the incubation time increased, the order degree also decreased, reaching the lowest order degree of 0.21 at 24 h (Fig. 3B, C). Based on previous research results, we found a peculiar phenomenon. After being cultured in pH 2.3 medium for 10 h, the WT strain showed an increase in GFP-D4 expression (i.e., the content of sterol analogs increased), but the order of membrane microdomains decreased (i.e., the proportion of membrane microdomains in the cell membrane decreased). This phenomenon was attributed to a significant change in the composition or structure of the membrane microdomains, which requires further evidence.

### Transcriptomic analysis probing relevant components associated with acid tolerance

**Fig. 4:**
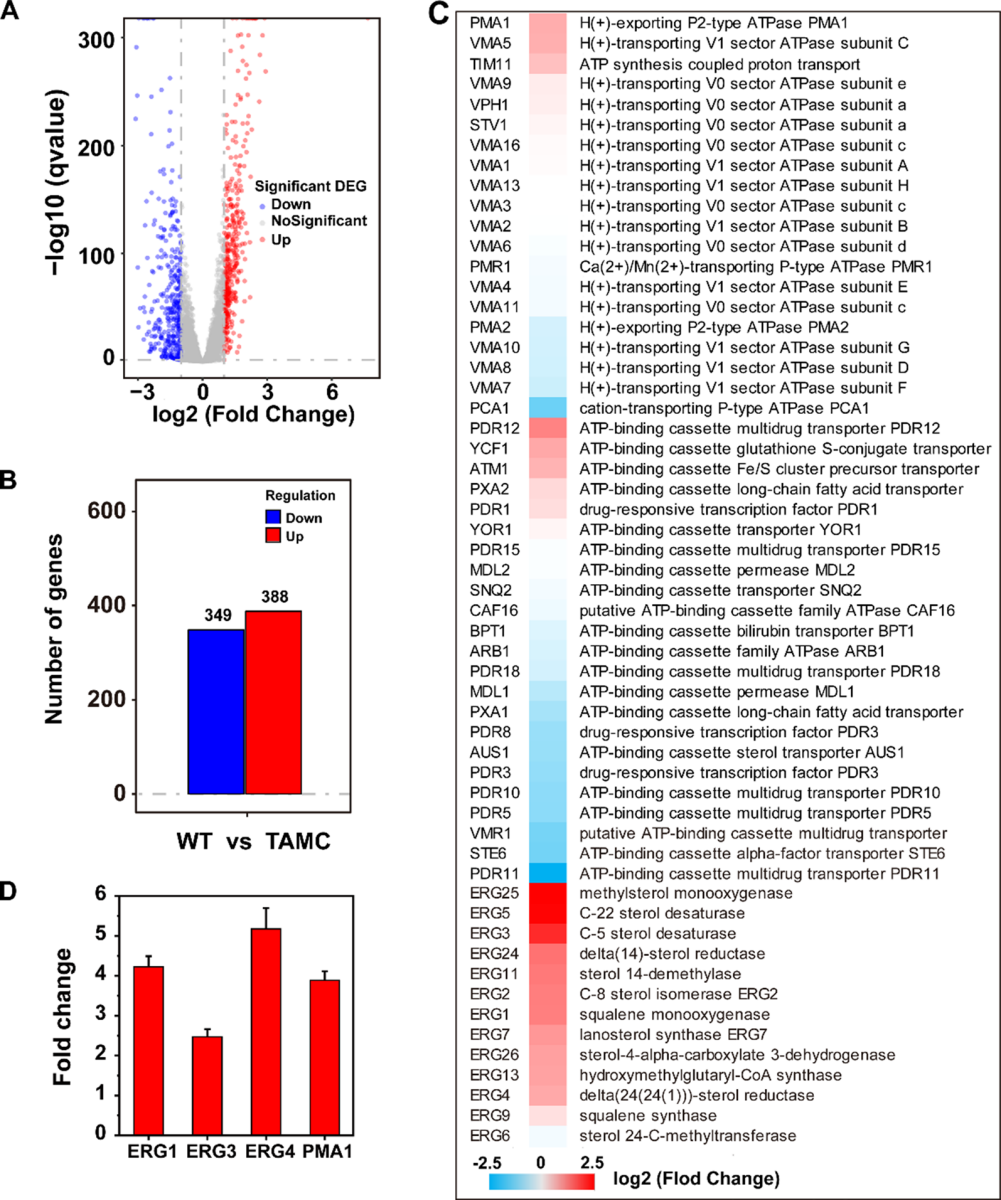
Transcriptional analysis of the TAMC strain. **A** Volcano plot demonstrates the genes with different expression levels. **B** Transcriptional levels of genes upregulated and downregulated in the TAMC strain compared with WT strain based on transcriptional analysis. **C** Significantly regulated genes of the TAMC strain. **D** Relative transcriptional levels of *ERG1*, *ERG3*, *ERG4*, and *PMA1*

To further explore the correlation between membrane microdomains and acid tolerance, differences in gene transcription between WT and TAMC strains were analyzed using transcriptomics. Comparative transcriptome data showed that the expression of 388 genes was significantly upregulated, whereas that of 349 genes was significantly downregulated (Fig. 4A, B). Similar to previous reports revealing that *PMA1* and *Pdr18* (ABC transporter) were involved in the acid resistance of yeast [18, 29], we observed significant changes in the expression of 20 H^+^-transporters and 23 ABC transporters, including *PMA1* in the TAMC strain compared with those in the WT strain (Fig. 4C). Further, the expression levels of the key genes involved in the ergosterol synthesis pathway, including *ERG1, ERG3,* and *ERG4,* as well as marker proteins for membrane microdomains, such as *PMA1*, increased significantly in the TAMC strain (Fig. 4C), consistent with the abovementioned visualization observation of GFP-D4 and membrane order analysis. At the same time, the expression level of some H^+^-transporters and ABC transporters decreased, which may be due to differences in the function and expression time of different transporters [30]. qRT-PCR analysis showed that the expression levels of *ERG1*, *ERG3*, *ERG4*, and *PMA1* in the TAMC strain were 4.2, 2.4, 5.1, and 3.7 times higher than those in the WT strain, respectively (Fig. 4D). Similar to our findings, several reports have also proposed the role of ergosterol synthesis in the acid tolerance of yeast [31–34]. For example, Guo et al. found that the accumulation of ergosterol is a protective mechanism for *S. cerevisiae* in response to organic acids, and ERG1 plays a key role in acid tolerance [31].

### Effects of membrane microdomain destruction on the acid tolerance of strains

**Fig. 5:**
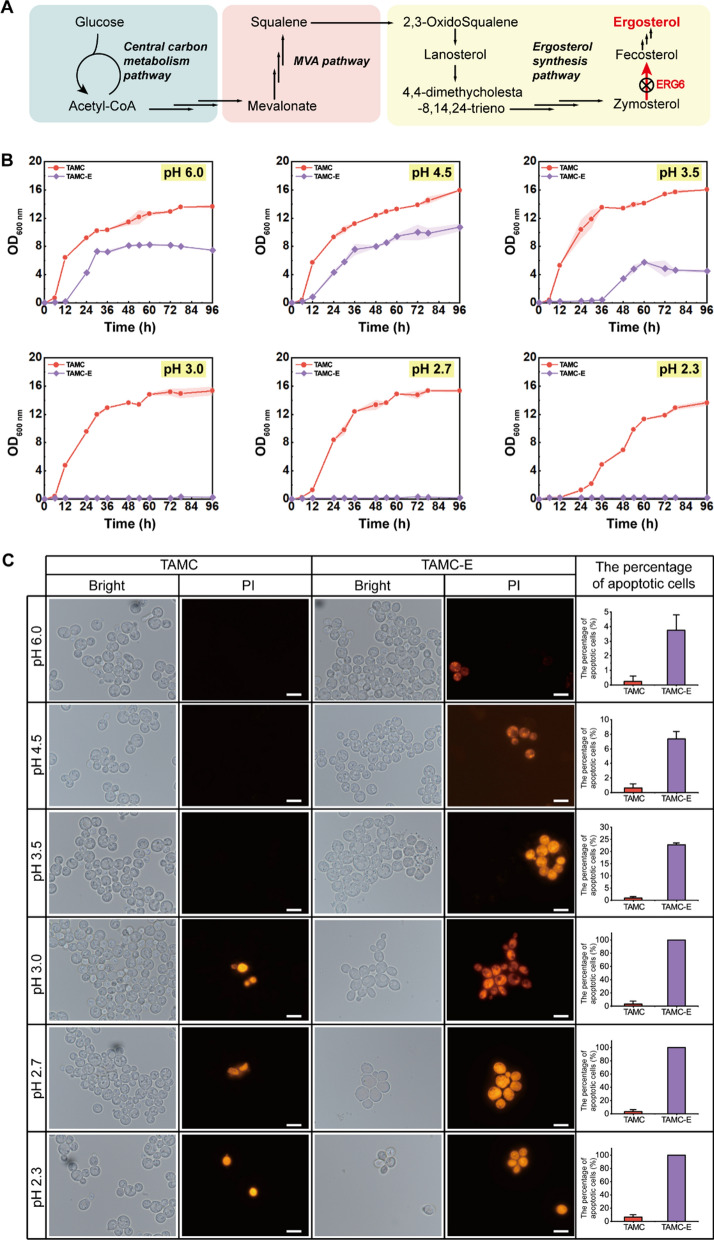
Effects of membrane microdomain destruction on TAMC and TAMC-E strains. **A** Schematic diagram of the ergosterol synthesis pathway. The cross indicates *ERG6* knockout. **B** Changes in biomass (OD_600 nm_) of TAMC and TAMC-E strains in YPD medium at different pH values. **C** PI staining of TAMC and TAMC-E strains in YPD medium at different pH values

Considering that ergosterol is an important component of membrane microdomains, this study attempted to inhibit ergosterol synthesis from suppressing the formation of membrane microdomains. First, ergosterol synthesis was successfully blocked by knocking out the Delta(24)-sterol C-methyltransferase encoding gene *ERG6* in TAMC strain to obtain the TAMC-E strain (Fig. 5A). Using L-malic acid, the pH of YPD medium was adjusted to six gradients of 6.0, 4.5, 3.5, 3.0, 2.7, and 2.3. As shown in Fig. 5B, the TAMC strain showed good growth status in different pH media throughout the cultivation process. Compared with TAMC strain, the TAMC-E strain showed decreased growth in pH 6.0 and 4.5 media, but its OD_600 nm_ value was maintained at ~ 10.0 during the stationary phase. However, cell growth was significantly inhibited when the pH drops < 3.5, with almost no growth. To verify these results, cell viability was further analyzed the via PI staining. TAMC and TAMC-E strains were first cultured in normal YPD medium for 24 h, and transferred to pH 6.0, 4.5, 3.5, 3.0, 2.7, and 2.3 media. After 48 h of culture, the apoptosis rate of the TAMC strains was < 6.4%, whereas that of TAMC-E cells reached 3.8%, 7.4%, 22.7%, 100%, 100%, and 100%, respectively (Fig. 5C). That is, when membrane microdomains were destroyed by knocking out *ERG6*, the strain could not tolerate the weak acid environment well, and a similar trend could also be observed in the wild-type strain with *ERG6* knockout (Additional file 1: Fig. S2). These results indicated that intact membrane microdomains are important for maintaining the acid-tolerance ability of the strains. Many previous studies have indicated that the aromatic acid transporter *ESBP6*, proton pump, or other ABC transporters are involved in the regulation of acid tolerance [12, 18, 19, 35]. However, they can only improve the tolerance of microorganisms to weak acids (pH 3.5–5.8). Combined with the analysis results, it was presumed that neglecting of the role of membrane microdomains makes it difficult to obtain strains with acid tolerance to pH of < 3.5. Therefore, to improve the acid tolerance of strains, in addition to strengthening H^+^-ATPase and transporters, it is also necessary to pay attention to the rational modification of membrane microdomains, which mainly consist of ergosterol, sphingomyelin, and scaffold proteins [21, 22].

### Regulation of membrane microdomains on H^+^-ATPase

**Fig. 6:**
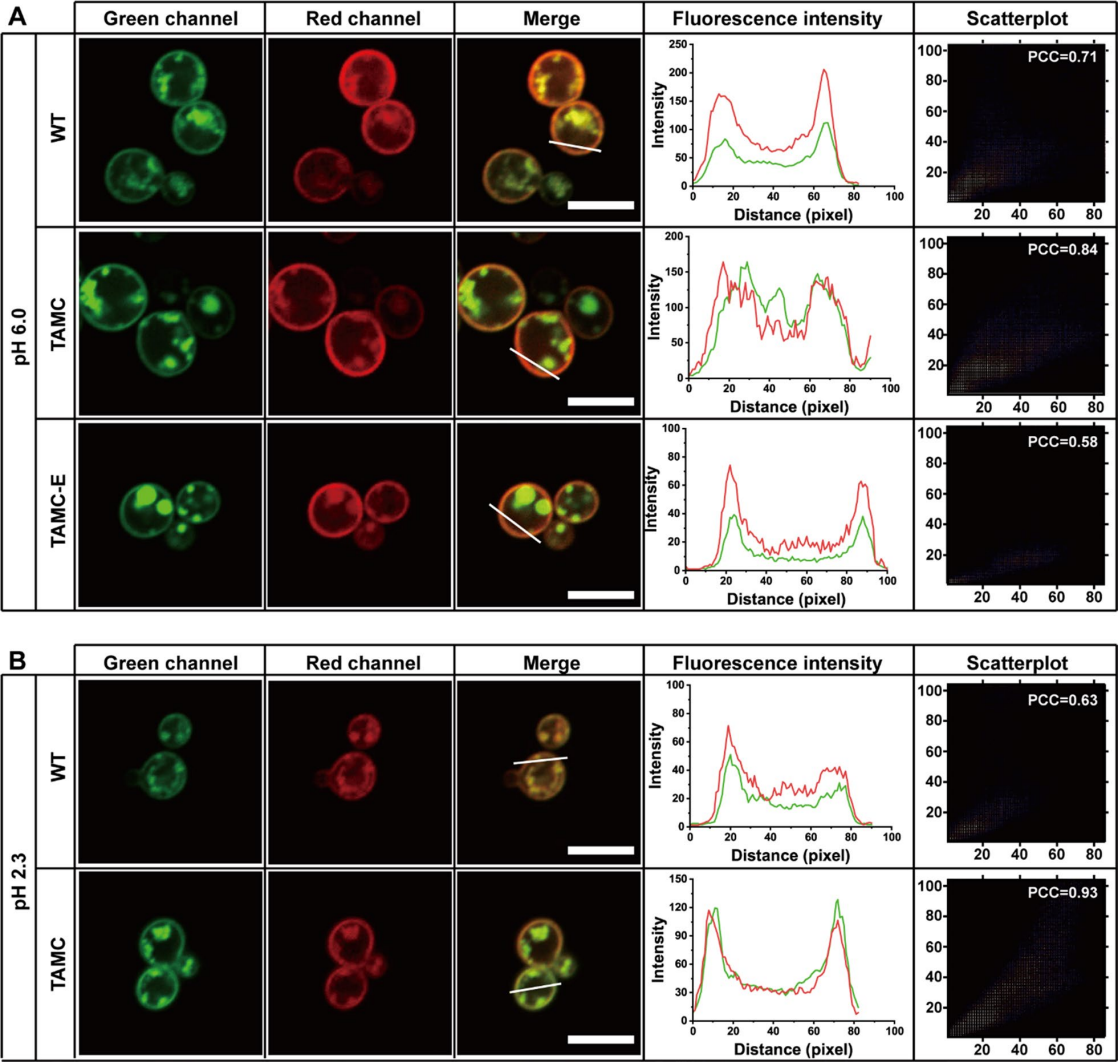
Colocalization of PMA1–GFP and PMP1–mCherry in WT, TAMC, and TAMC-E strains in different YPD media. **A** Colocalization analysis of PMA1–EGFP and PMP1–mCherry in WT, TAMC, and TAMC-E strains at pH 6.0. **B** Colocalization analysis of PMA1–EGFP and PMP1–mCherry in WT, TAMC, and TAMC-E strains at pH 2.3. The first three columns indicate the confocal dual-channel imaging and merging images of PMA1–EGFP and PMP1–mCherry. The fourth column is the plot of pixel intensity along the white line from left to right of each plot, with colors in merged images. The fifth column represents the scattergram of the first and second lines. Scale bar, 5 µm

PMA1 H^+^-ATPase plays an essential role in improving the ability of strains to tolerate weak acids [19], and PMP1, a 38-residue plasma membrane protein, could regulate the H^+^-ATPase activity [36]. In this study, using plasmid pY15, PMA1 and PMP1 were expressed as fusion proteins with GFP and mCherry (red fluorescent protein), respectively. As shown in Fig. 6A confocal imaging revealed that PMA1-GFP and PMP1-mCherry appeared on plasma membranes, showing good colocalization in the WT strain at pH 6.0. Pearson’s correlation coefficient (PCC), determined using a colocalization finder via ImageJ according to the previously reported method [37], was used to quantitatively analyze the correlation between PMA1 and PMP1. Results showed that the PCC of PMA1-GFP and PMP1-mCherry in the WT strain was 0.71, which was lower than that in the TAMC strain (PCC = 0.84). When the strains were incubated in pH 2.3 medium, the PPC of the WT strain decreased to 0.63, while that of TAMC strain increased to 0.93. Regarding the TAMC-E strain, the colocalization degree of PMA1-GFP and PMP1-mCherry decreased evidently, with a PCC of only 0.58 in pH 6.0 medium (Fig. 6B). Notably, no living cells were detected when the TAMC-E strain was transferred into pH 2.3 medium, even after 5 min of culture with pH 2.3 medium (Additional file 1: Fig. S3). The above results indicated that complex formation of PMA1 and PMP1 are key to acid resistance, and membrane microdomains play an indispensable role in promoting the complex formation of PMA1 and PMP1.

Membrane microdomains can serve as platforms for protein docking and are closely related to physiological processes such as membrane signal transduction and protein sorting [38]. In human astrocytes, membrane microdomains (also known as lipid rafts) may provide a physical platform for the localization and processing of amyloid precursor protein [39]. Similarly, the membrane microdomains in plant cells could promote the formation of AtHIR1-associated immune complexes upon perception of the pathogen [40]. Combining previous research and current study findings, it can be speculated that membrane microdomains provide a physical platform for forming of H^+^-ATPase complexes to maintain intracellular H^+^ homeostasis, thereby promoting cells to better resist acidic environments.

## Conclusion

Based on visual observation and omics analysis, our study showed that membrane microdomains play a crucial role in acid tolerance in *S. cerevisiae* TAMC. In particular, membrane microdomains can provide the platform for acid tolerance-related elements, such as H^+^-ATPase. These results shed new light on the acid-tolerance mechanism of yeast and construction of acid-resistant chassis cells.

## Materials and methods

### Strains, plasmids and reagents

The *Escherichia coli* JM109 strain was used for genetic experiments, *S. cerevisiae* TAMC was selected as the experimental strain, and *S. cerevisiae* CEN.PK2-1C served as the control strain. All strains and plasmids used in this study are listed in Table 1. FM4-64 [N-(3-triethylammomiumpropyl)4-(P-diethylaminophenylhexatrienyl)] (PI; ThermoFisher Scientific, USA) and di-4- ANEPPDHQ (Invitrogen, USA) were used from dimethyl sulfoxide (DMSO) stock solution (5 mM), and propidium iodide (PI, Solarbio, China) was used from phosphate-buffered saline (PBS) stock solution (5 mM). The stock solution was diluted with yeast extract peptone dextrose (YPD) medium for use, and the final DMSO concentration was ≤ 0.1% (v/v) for all analyses.

**Table 1:** Strains and plasmids used in this study

	Description	Source
Strains		
*E. coli* JM109	*E. coli*, for plasmid construction	Lab stock
WT	CEN.PK2-1C, MATa; his3D1; leu2-3_112; ura3-52; trp1-289; MAL2-8c; SUC2	Lab stock
TAMC	CEN.PK2-1C derivate, which can tolerate a low pH of 2.3	[15]
WT-D4	WT derivate, expressing the plasmid pY15-GFP-D4	This study
TAMC-D4	TAMC derivate, expressing the plasmid pY15-GFP-D4	This study
TAMC-E	TAMC derivate, *ΔERG6*	This study
WT-E	WT derivate, *ΔERG6*	This study
WT-PP	WT derivate, expressing the plasmid pY15-PMP1-mCherry-PMA1-EGFP	This study
TAMC-PP	TAMC derivate, expressing the plasmid pY15-PMP1-mCherry-PMA1-EGFP	This study
Plasmids		
pY15	Amp, LEU2, CEN/ARS, *E. coli-S. cerevisiae* shuttle vector	Lab stock
pY15-GFP-D4	pY15 derivate, inserting P_TEF1_-GFP-D4-T_CYC1_	This study
pY15-PMP1-mCherry-PMA1-EGFP	pY15 derivate, inserting P_TEF1_-PMP1-mCherry-T_CYC1_ and P_GAP_-PMA1-EGFP-T_AOX1_	This study

### Culture media and conditions

All *E. coli* strains were cultured in Luria–Bertani (LB) medium or on LB agar plates at 37 °C. *S. cerevisiae* was generally cultured in the YPD medium (20 g/L glucose, 10 g/L yeast powder, and 20 g/L tryptone) at 30 °C. The pH of YPD medium was adjusted by L-malic acid to 6.0, 4.5, 3.5, 3.0, 2.7, and 2.3. To screen for recombinant *S. cerevisiae* strains, the yeast nitrogen base medium lacking appropriate nutrients was used. To quantify cell growth, a single colony was inoculated into a 5-mL YPD medium and cultured for 24 h at 30 °C. A 1% seed culture was transferred to a 250-mL flask containing 30 mL YPD medium at different pH values and cultured for 96 h at 30 °C. Finally, OD_600 nm_ was quantified at specific intervals.

### Construction of plasmids and recombinant strains

PrimeSTAR max premix DNA polymerase (TaKaRa, Japan) was used for gene amplification. Rapid Taq Master Mix (Vazyme, China) was used for colony polymerase chain reaction (PCR) verification. All terminators or promoters were amplified using the genome of *S. cerevisiae* 288C as the template, and the plasmid pY15 was used to construct gene expression cassettes. All fragments and plasmids were verified via sequencing before transformation. Homologous recombination was performed in *S. cerevisiae* using the CRISPR–Cas9 or CRISPR–loxP system to achieve gene knockout or gene expression cassette integration [41]. Using the LiAc/PEG/ssDNA method, the plasmid DNA or PCR fragments were transformed into *S. cerevisiae* to construct the desired recombinant strains.

### Staining, image acquisition and analysis

For FM4-64 staining, strains were incubated with 8 µM FM4-64 for 5 min in culture medium. For di-4-ANEPPDHQ staining, concentrated yeast strains were incubated in 5 μM di-4-ANEPPDHQ solution (diluted with YPD medium) for 10 min on ice and then washed at least 3 times with cold YPD medium. The 488-nm laser was used to excite the samples, and the detection ranges of the two channels were set at 500–580 and 620–750 nm, respectively.

Generalized polarization (GP) images and GP values were generated and calculated according to the previously published methods [27, 42]. Upon multiplying the GP value by the intensity value of each pixel, the hue-saturation-brightness (HSB) value can be generated. For FM4-64 and di-4-ANEPPDHQ staining, a Leica TCS SP8 microscope fitted with a 60 × oil-immersion objective was used. For PI staining, concentrated yeast strains were incubated in PBS (pH 6.0) at 30 °C for 10 min. Fluorescence imaging was performed under a Nikon C-HGF microscope fitted with a 100 × oil immersion objective. Apoptotic cells stained with PI were observed using a TRITC fluorescent filter.

### Transcriptome analysis

Wild type (WT) and TAMC strains were cultured in YPD medium (pH 6.0) until the mid-log phase and placed in liquid nitrogen to block the metabolism. Six replicates were set for each strain, and the samples were sent to Genewiz Biotechnology Co., Ltd. (Suzhou, China) for further analyses. Transcriptomics analysis was accomplished using Illumina HiSeq-4000 based on the services of the RNA-seq Quantification Library at Genewiz Biotechnology. RNA-seq data were assembled and analyzed by comparing with the translational region of the annotated DNA sequence data of reference (NCBI:txid 559,292) using HiSat2 and HTSeq [43, 44]. The estimation of fold change and other statistical analyses were performed using DESeq.2 [45]. Annotation classification was performed according to the Kyoto Encyclopedia of Genes and Genomes public database, and enrichment analysis was conducted using the phyper function in R software. The *p* value was calculated. False discovery rate correction was applied to the *p* value to obtain *q* value, where *q* ≤ 0.05 was considered significantly enriched.

### Scanning electron microscopy (SEM) analysis

Cultured cells were washed thrice with PBS, and fixed at 4 °C for 2 h with 3% glutaraldehyde precooled at 4 °C. After the fixative was sucked out, cells were washed twice with PBS, fixed at 4 °C for 1 h, precooled with 1% osmic acid at 4 °C, and oaked with PBS thrice. Dehydration with acetone and isoamyl acetate (1:1) was performed for 10 min, followed by dehydration with a series of gradient alcohols (30%, 50%, 70%, 80%, 90%, and 100%). Finally, the samples were dehydrated using a critical point dryer.

### Quantitative reverse transcription PCR (qRT–PCR) analysis

Yeast cells in the mid-log phase were collected and centrifuged at 8000 ×*g* for qRT-PCR analysis. Total RNA was purified using the Yeast Processing Reagent (Takara, Japan) and RNAiso Plus (Takara, Japan). cDNA synthesis was conducted using PrimeScript™ RT reagent kit with gDNA Eraser (Takara, Japan). qRT-PCR was performed using TB Green^®^ Premix Ex Taq™ II (Takara, Japan) via a LightCycler 480 II Real-time PCR instrument (Roche Applied Science, Germany). The primers for qRT-PCR are listed in Additional file 1: Table S1.

### Statistical analysis

Three independent replicates were performed for all experiments, except for the parallel analysis of omics, which used six replicates. Statistical analysis was performed using *t*-test. *p*-values of < 0.05 were considered to indicate statistical significance (*p < 0.05; **p < 0.01).

### Supplementary Information

**Additional file 1: Table S1.** Primers used in this study. **Figure S1.** Scanning electron microscope images of strains WT and TAMC in pH 6.0 (A) and pH 2.3 (B) medium. Scale bar = 5 µm. **Figure S2.** Effects of membrane microdomain destruction on WT and WT-E strains. Changes in biomass (OD_600 nm_) of WT and WT-E strains in YPD medium at different pH values. **Figure S3.** The co-localization of PMA1-GFP and PMP1-mCherry of strain TAMC-E in YPD medium of pH 2.3. Scale bar, 5 µm.

## Data Availability

The data and materials used in this study are available from the corresponding authors on reasonable request.
