# Supplementary material for: Analysis of acid-tolerance mechanism based on membrane microdomains in Saccharomyces cerevisiae
Source: Microb Cell Fact. 2023 Sep 13;22:180. doi: 10.1186/s12934-023-02195-y (PMC10498586; doi:10.1186/s12934-023-02195-y)
Supplement: Supplementary file 1 — Additional file 1: Table S1. Primers used in this study. Figure S1. Scanning electron microscope images of strains WT and TAMC in pH 6.0 (A) and pH 2.3 (B) medium. Scale bar = 5 µm. Figure S2. Effects of membrane microdomain destruction on WT and WT-E strains. Changes in biomass (OD600 nm) of WT and WT-E strains in YPD medium at different pH values. Figure S3. The co-localization of PMA1-GFP and PMP1-mCherry of strain TAMC-E in YPD medium of pH 2.3. Scale bar, 5 µm. [file 12934_2023_2195_MOESM1_ESM.docx]

# Additional files

**Analysis of** **acid-tolerance mechanism based on membrane microdomains in *Saccharomyces cerevisiae***

Xueqin Lv^a, b, c^, Ke Jin^b, c^, Yu Yi ^b^, Lingang Song ^b^, Xiang Xiu ^a, b^, Yanfeng Liu ^a, b^, Jianghua Li ^a, b^, Guocheng Du ^a, b^, Jian Chen^a, b, c^, Long Liu ^a, b, c, d *^

^a^ Key Laboratory of Carbohydrate Chemistry and Biotechnology, Ministry of Education, Jiangnan University, Wuxi 214122, China.

^b^ Science Center for Future Foods, Jiangnan University, Wuxi 214122, China.

^c^ Yixing Institute of Food Biotechnology Co., Ltd, Yixing 214200, China

^d^ Food Laboratory of Zhongyuan, Jiangnan University, Wuxi 214122, China.

**Table S1 Primers used in this study**

| **Primers** | **Sequence (5'-3')** |
| --- | --- |
| ERG1QPCR-F | ggaattcaaagcccacttga |
| ERG1QPCR-R | taacgtgaccgtgcatagga |
| ERG3QPCR-F | tagaaggttctacgggcagg |
| ERG3QPCR-R | acgaggatggttgaaaatcg |
| ERG4QPCR-F | tcgggtttccactgctaatg |
| ERG4QPCR-R | cccgggcaacgtatagtag |
| PMA1QPCR-F | tcggttggtggtctgaaaac |
| PMA1QPCR-R | ttccttcattggcttaccg |


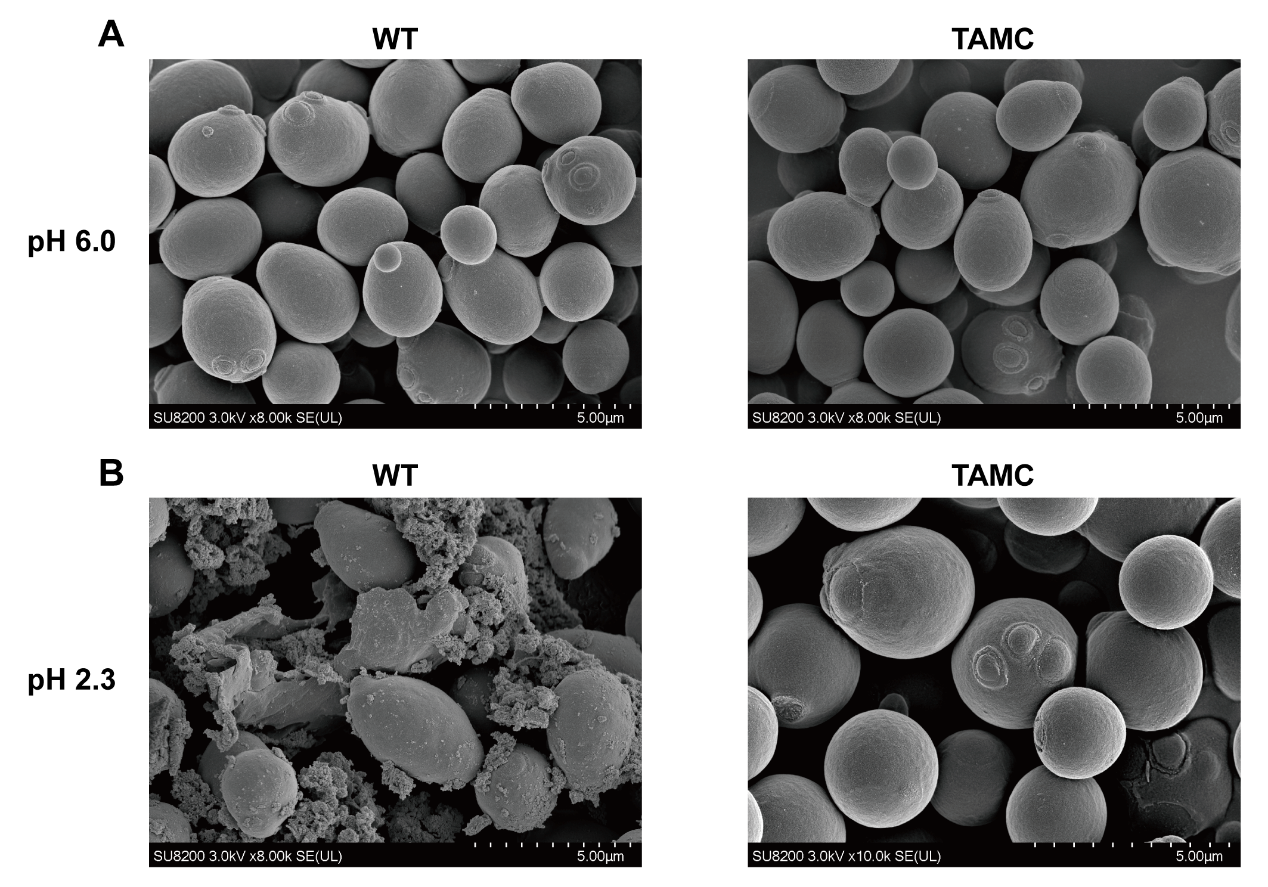


**Figure S1.** Scanning electron microscope images of strains WT and TAMC in pH 6.0 (A) and pH 2.3 (B) medium. Scale bar= 5 µm.

**
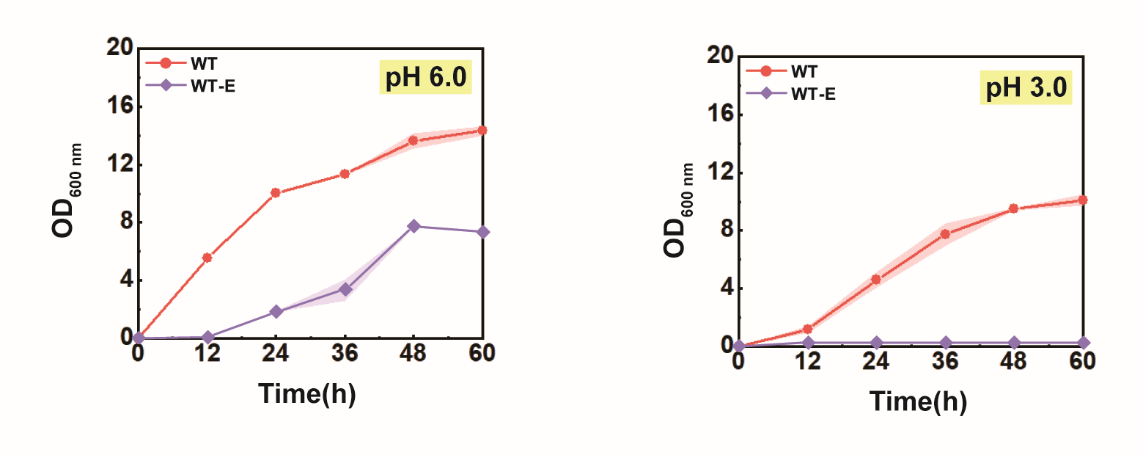
**

**Figure S2.** Effects of membrane microdomain destruction on WT and WT-E strains. Changes in biomass (OD_600 nm_) of WT and WT-E strains in YPD medium at different pH values.

**
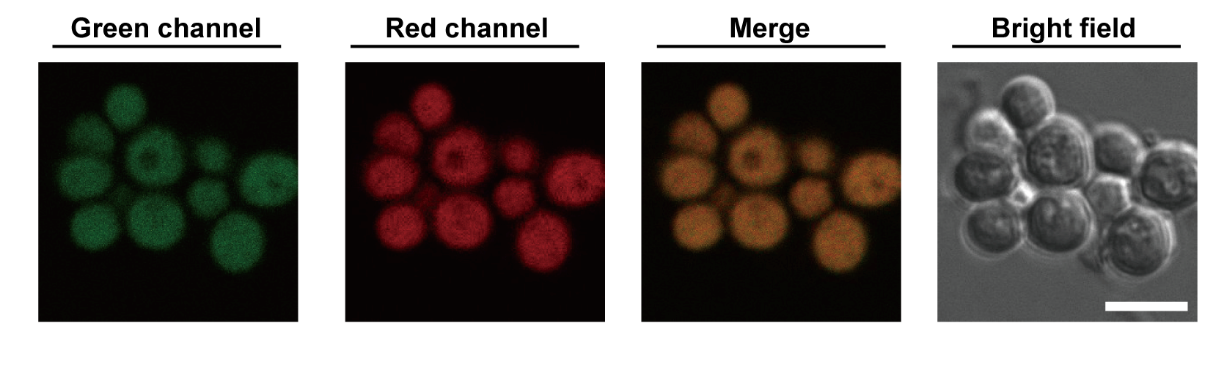
**

**Figure S3.** The co-localization of PMA1-GFP and PMP1-mCherry of strain TAMC-E in YPD medium of pH 2.3. Scale bar, 5 µm.
